# An external validation study reporting poor correlation between the claims-based index for rheumatoid arthritis severity and the disease activity score

**DOI:** 10.1186/s13075-015-0599-0

**Published:** 2015-04-13

**Authors:** Rishi J Desai, Daniel H Solomon, Michael E Weinblatt, Nancy Shadick, Seoyoung C Kim

**Affiliations:** Division of Pharmacoepidemiology and Pharmacoeconomics, Department of Medicine, Brigham and Women’s Hospital & Harvard Medical School, 1620 Tremont Street, Boston, 02120 MA USA; Division of Rheumatology, Immunology and Allergy, Brigham and Women’s Hospital, 75 Francis Street, Boston, 02125 MA USA

## Abstract

**Introduction:**

We conducted an external validation study to examine the correlation of a previously published claims-based index for rheumatoid arthritis severity (CIRAS) with disease activity score in 28 joints calculated by using C-reactive protein (DAS28-CRP) and the multi-dimensional health assessment questionnaire (MD-HAQ) physical function score.

**Methods:**

Patients enrolled in the Brigham and Women’s Hospital Rheumatoid Arthritis Sequential Study (BRASS) and Medicare were identified and their data from these two sources were linked. For each patient, DAS28-CRP measurement and MD-HAQ physical function scores were extracted from BRASS, and CIRAS was calculated from Medicare claims for the period of 365 days prior to the DAS28-CRP measurement. Pearson correlation coefficient between CIRAS and DAS28-CRP as well as MD-HAQ physical function scores were calculated. Furthermore, we considered several additional pharmacy and medical claims-derived variables as predictors for DAS28-CRP in a multivariable linear regression model in order to assess improvement in the performance of the original CIRAS algorithm.

**Results:**

In total, 315 patients with enrollment in both BRASS and Medicare were included in this study. The majority (81%) of the cohort was female, and the mean age was 70 years. The correlation between CIRAS and DAS28-CRP was low (Pearson correlation coefficient = 0.07, *P* = 0.24). The correlation between the calculated CIRAS and MD-HAQ physical function scores was also found to be low (Pearson correlation coefficient = 0.08, *P* = 0.17). The linear regression model containing additional claims-derived variables yielded model R^2^ of 0.23, suggesting limited ability of this model to explain variation in DAS28-CRP.

**Conclusions:**

In a cohort of Medicare-enrolled patients with established RA, CIRAS showed low correlation with DAS28-CRP as well as MD-HAQ physical function scores. Claims-based algorithms for disease activity should be rigorously tested in distinct populations in order to establish their generalizability before widespread adoption.

## Introduction

One of the most important confounders in observational studies of patients with rheumatoid arthritis (RA) is disease severity. Owing to large size and relative ease of access, health-care utilization databases have been increasingly used to study various treatment outcomes in RA [1-4]. However, clinical disease activity markers are not available in these databases, and hence studies conducted using these data are prone to residual confounding by disease severity. To address this problem, Ting *et al*. [5] developed an algorithm to create a claims-based index for rheumatoid arthritis severity (CIRAS) by using numerous variables from claims. In their original article, Ting *et al*. [5] used RA records-based index of severity (RARBIS), which was constructed by using ratings by a Delphi panel on potential markers of RA severity commonly found in medical charts, to demonstrate the validity of CIRAS and reported moderate correlation between the medical records and claims-based indices.

Despite being commonly used in RA observational research [6-8], CIRAS has not been validated against a clinical marker of RA severity until now. RA severity is a complex concept that depends on a combination of disease activity, physical function impairment, and physical damage to the joints. Clinically accepted measures for accurately determining RA severity that include all of these aspects are scarce. However, the disease activity score in 28 joints calculated by using C-reactive protein (DAS28-CRP) [9] is commonly used to evaluate treatment success and to guide treatment selection in patients with RA [10]. Therefore, in the absence of a standard clinical measure for RA severity, we selected the disease activity measure DAS28-CRP to validate the claims-based severity measure CIRAS in this external validation study using data from the Brigham and Women’s Hospital Rheumatoid Arthritis Sequential Study (BRASS) linked to Medicare claims. Furthermore, we examined the correlation between the multi-dimensional health assessment questionnaire (MD-HAQ) [11] physical function scores and CIRAS.

## Methods

The BRASS registry is a single-center, prospective, observational cohort of 1,350 patients with a rheumatologist-verified diagnosis of RA. For the subjects enrolled in this registry, data on patient-reported items, including demographics, lifestyle factors, medication use, and quality-of-life scales, as well as physician-reported items such as DAS28-CRP, extra-articular manifestations, and medication changes are collected during annual follow-up visits. For this study, we identified BRASS patients who were also enrolled in Medicare between 2006 and 2010, and linked their data from these two sources. Of these subjects, we further identified those with at least one valid DAS28-CRP measurement in BRASS after 365 days of continuous enrollment in Medicare. The algorithm proposed by Ting *et al*. [5] was implemented by using Medicare claims data in the period of 365 days immediately prior to the DAS28-CRP measurement date to calculate the CIRAS for these patients. Pearson correlation coefficients between the calculated CIRAS and DAS28-CRP were calculated. We also analyzed MD-HAQ physical function scores measured on the same day as DAS28-CRP for these patients and calculated Pearson correlation coefficients between the calculated CIRAS and MD-HAQ. Personal identifiers were removed from the dataset before the analysis to protect subject confidentiality. Patient informed consent was, therefore, not required. This study was approved by the Brigham and Women’s Hospital’s Institutional Review Board.

Furthermore, we identified several other potential predictors of RA severity, which were not part of the original CIRAS, from medical and pharmacy claims in a subset of patients who had Medicare part D enrollment for the 365-day period prior to the DAS28 measurement date in order to improve the algorithm for CIRAS. These variables included rheumatoid lung involvement, hand surgery, tuberculin test ordered, and anti-cyclic citrullinated peptide (CCP) test ordered, steroid use, opioid use, non-steroidal anti-inflammatory drug (NSAID) use, number of non-biologic disease-modifying anti-rheumatoid drugs (DMARDs) used, and biologic DMARD use. A multivariable linear regression model was built by using DAS28-CRP as the outcome and these claims-derived variables as predictors. Adjusted correlations between the predictors and the outcome were reported as partial R^2^ values. Full model R^2^ was reported as a measure of the overall performance of this model.

## Results

We located 368 patients who were enrolled in both BRASS and Medicare. We then excluded 53 patients who did not have at least one valid DAS28-CRP measured in BRASS after 365 days of continuous enrollment in Medicare, leaving 315 patients with sufficient baseline data for calculation of CIRAS. Of these 315 patients, the majority (81%) were females. The mean (standard deviation) age of the cohort was 70 (10) years. The median (interquartile range) DAS28-CRP and CIRAS were 3.3 (2.3 to 4.6) and 4.4 (3.7 to 5.1), respectively. Other patient characteristics used for CIRAS calculation are summarized in Table 1. The correlation between the calculated CIRAS and DAS28-CRP was found to be poor (Pearson correlation coefficient = 0.07, *P* = 0.24). The correlation between the calculated CIRAS and MD-HAQ physical function scores was also found to be low (Pearson correlation coefficient = 0.08, *P* = 0.17).

**Table 1:** **Characteristics of rheumatoid arthritis patients included in the external validation study**

**Variable**	**Number (percentage)**
	**(Total number = 315)**
Components of the algorithm for calculating CIRAS	
Age in years, mean (SD)	70 (10)
Females	255 (81.0)
Inflammatory marker tests ordered	230 (73.0)
Rehabilitation visits (physical or occupational therapy visits)	75 (23.8)
Rheumatoid factor test ordered	17 (5.4)
Felty’s syndrome	2 (0.6)
Platelet counts ordered	
None	47 (14.9)
1	24 (7.6)
2	33 (10.5)
3	35 (11.1)
≥4	176 (55.9)
Number of chemistry panels ordered	
None	76 (24.1)
1	37 (11.7)
2	52 (16.5)
3	31 (9.8)
4	25 (7.9)
≥5	94 (29.8)
Number of rheumatologist visits	
None	51 (16.2)
1 to 4	220 (69.8)
>4	44 (14.0)
Rheumatoid arthritis severity variables	
CIRAS, mean (SD)	4.4 (1.2)
DAS28-CRP, mean (SD)	3.5 (1.5)
MD-HAQ physical function score, mean (SD)	2.5 (1.9)

Data are from the Brigham and Women’s Hospital Rheumatoid Arthritis Sequential Study linked with Medicare claims (2006 to 2010). CIRAS, claims-based index for rheumatoid arthritis severity; DAS28-CRP, disease activity score in 28 joints calculated by using C-reactive protein; MD-HAQ, multi-dimensional health assessment questionnaire; SD, standard deviation.

Furthermore, we identified a subgroup of 119 patients who had at least 1 year of Medicare part D enrollment immediately prior to the DAS28-CRP measurement date. The linear regression model containing additional claims-derived variables along with the variables originally proposed by Ting *et al*. [5] yielded model R^2^ of 0.23, suggesting limited ability of this model to explain variation in DAS28-CRP. Among some of the most influential predictors in this model were biologic DMARD use, opioid use, tuberculin test ordered, and number of non-biologic DMARDs used in the prior year (Table 2).

**Table 2:** **Adjusted correlations between additional claims-based variables related to rheumatoid arthritis severity and DAS28-CRP**

**Variable**	**Partial** **R** ^**2**^
Original variables included in the algorithm to calculate CIRAS^a^	
Age	0.001
Gender	0.001
Inflammatory marker tests ordered	0.004
Rehabilitation visits (physical or occupational therapy visits)	0.0001
Rheumatoid factor test ordered	0.003
Platelet counts ordered	0.03
Number of chemistry panels ordered	0.001
Number of rheumatologist visits	0.004
Additional medical claims-based variables	
Rheumatoid lung involvement	0.005
Hand surgery	0.006
Tuberculin test ordered	0.05
Anti-CCP test ordered	0.005
Additional pharmacy claims-based variables	
Steroid use	0.004
Opioid use	0.05
NSAID use	0.0003
Number of non-biologic DMARDs used	0.03
Biologic DMARD use	0.05
Model R^2^	0.23

Data are from the Brigham and Women’s Hospital Rheumatoid Arthritis Sequential Study linked with Medicare claims (2006 to 2010). ^a^A subsample of patients with pharmacy claims available (119 out of 315) was used to calculate these R^2^ values. In this subsample, none of the patients had a Felty’s syndrome diagnosis. Therefore, that variable is not reported in this table. All of the variables were measured in the period of 365 days immediately prior to the DAS28-CRP (disease activity score in 28 joints calculated by using C-reactive protein) measurement date. CIRAS, claims-based index for rheumatoid arthritis severity; CCP, cyclic citrullinated peptide; DMARD, disease-modifying anti-rheumatic drug; NSAID, non-steroidal anti-inflammatory drug.

## Discussion

In this validation study using data from an external cohort of Medicare-enrolled patients with an established diagnosis of RA, the previously published algorithm to approximate RA severity by using claims-based variables had poor correlation with DAS28-CRP and MD-HAQ. Adding more variables derived from both medical and pharmacy claims as predictors in a linear regression model did not substantially improve the performance of this algorithm in predicting DAS28-CRP.

Several potential differences between this external validation study and the original study in which Ting *et al*. [5] developed CIRAS may explain the poor performance of CIRAS in this cohort. First, it must be noted that CIRAS was validated against a medical records-based RA severity index (RARBIS) in the original study and that the correlation between the two indices was found to be moderate (Spearman correlation coefficient = 0.51). RARBIS itself has been shown to correlate only moderately with DAS28 (Spearman correlation coefficient = 0.41) [12]. The majority of the clinical parameters measured through RARBIS, including patients’ functional status, arthritis flares, x-ray results, and laboratory results, are not captured in claims and hence in CIRAS. Therefore, the poor performance of CIRAS against DAS28-CRP may simply reflect the inability to account for these important clinical parameters. Next, important differences between the current cohort and the CIRAS derivation cohort, including sizable gender differences (81% versus 9% females), differences in the disease activity, and differences in health-care utilization patterns, may help explain the poor performance of CIRAS in this validation cohort.

CIRAS has been used in observational studies of RA treatments in the past mainly to control for confounding by disease activity. Two prior studies used CIRAS as a covariate in their regression models for the outcome [6,7]. Another study used CIRAS as one of the variables for prediction of a disease risk score (infection score) and stratified analysis based on this disease risk score to account for measured confounding [8]. Findings from our study show poor correlation between CIRAS and DAS28-CRP (RA activity measure, which often drives treatment selection) as well as MD-HAQ (patient physical function score, which may be indicative of frailty and hence may be an important confounder). These findings suggest that CIRAS may not accurately approximate disease activity or frailty in observational studies of RA treatments using insurance claims data. Given this poor correlation between CIRAS and important confounders unmeasured in health-care claims data, future research should be considered to critically evaluate the benefit of using CIRAS as a tool for confounding control.

Another important contribution of our study is that it highlights the importance of external validation of claims-based algorithms. Two prior studies have attempted to build algorithms predicting RA severity. Wolfe *et al*. [13] used data on the type and number of DMARDs used by the patients in the National Data Bank for Rheumatic Diseases to predict their RA severity and found suboptimal performance of these variables in predicting RA severity as measured by a patient activity scale. Baser *et al*. [14] used Veterans Health Administration claims to build a severity index for rheumatoid arthritis (SIFRA) and reported moderate correlations with the CIRAS. Before widespread adoption of these indices, broad testing is critical to determine their appropriateness in different databases.

## Conclusions

Our study reported a low correlation between the previously proposed CIRAS and DAS28-CRP as well as MD-HAQ physical function scores, suggesting that CIRAS may not approximate RA disease activity or frailty reliably in observational cohorts. Claims-based algorithms for clinical disease activity should be rigorously tested in distinct populations in order to establish their generalizability.

## Abbreviations

BRASS: Brigham and Women’s Hospital Rheumatoid Arthritis Sequential Study
CIRAS: claims-based index for rheumatoid arthritis severity
DAS28-CRP: disease activity score in 28 joints calculated by using C-reactive protein
DMARD: disease-modifying anti-rheumatic drug
MD-HAQ: multi-dimensional health assessment questionnaire
RA: rheumatoid arthritis
RARBIS: rheumatoid arthritis records-based index of severity
